# Efficacy of Erector Spinae Plane (ESP) Block for Non-cardiac Thoracic and Upper Abdominal Surgery: A Single Institute Comparative Retrospective Case Series

**DOI:** 10.7759/cureus.58926

**Published:** 2024-04-24

**Authors:** Zasmine Hymes-Green, Erin L LaGrone, Jacelyn E Peabody Lever, Joel Feinstein, Paul D Piennette, Prentiss Lawson, Jason B Gerlak, Christopher A Godlewski, Brandon Brooks, Promil Kukreja

**Affiliations:** 1 Anesthesiology and Perioperative Medicine, University of Alabama at Birmingham (UAB), Birmingham, USA; 2 School of Medicine, University of Alabama at Birmingham (UAB), Birmingham, USA

**Keywords:** peri-operative analgesia, enhanced recovery pathway, postoperative pain, oral morphine equivalents, erector spinae plane (esp) block

## Abstract

Introduction

Erector spinae plane (ESP) block was first introduced for the management of thoracic pain but has become increasingly popular for the treatment of abdominal surgical pain. Previous studies have shown the ESP block can be easily adapted to abdominal procedures at the corresponding dermatome level and provide postoperative analgesia. Though the versatility, simplicity, and safety of the ESP block have been demonstrated, there is a gap in the literature regarding its comparison between thoracic and abdominal surgeries. This study aims to evaluate the efficacy of the ESP block in treating acute postoperative pain in patients undergoing thoracic and abdominal surgeries.

Methods

This retrospective study included 50 patients in the non-cardiac thoracic surgery group (bilateral breast mastectomy with reconstruction) and 50 patients in the abdominal surgery group (robotic or laparoscopic sleeve gastrectomy). Data was obtained via the acute pain service records at a tertiary care center from 2018 to 2022. All patients received bilateral ESP blocks, performed under ultrasound guidance. Various parameters were evaluated including oral morphine equivalents (OMEs) and visual analog scale (VAS) scores during post-anesthesia care unit (PACU), 6, 12, and 24 hours postop. The use of abortive antiemetic medications within 24 hours was also measured to evaluate the incidence of nausea and vomiting. The results were analyzed and compared. No control group is included, as all patients at our institution receive a peripheral nerve block as a part of the institution's enhanced recovery pathway (ERP).

Results

This retrospective study included 50 patients in the non-cardiac thoracic surgery group (bilateral breast mastectomy with reconstruction) and 50 patients in the abdominal surgery group (robotic or laparoscopic sleeve gastrectomy). Compared to the thoracic group, the abdominal group had a statistically higher VAS score in PACU with mean difference (MD) 1.3 VAS, 95% confidence interval (CI) 0.03-2.56, p-value 0.0443, statistically higher OME consumption in the PACU (difference 13.35 OME, 95% CI 4.97-21.73, p-value 0.0003), and required significantly more antiemetic pharmacotherapy (mean 1.4 antiemetics administered, 95% CI 0.84-2.04, p-value <0.0001). Despite the abdominal group having more OME utilization in the PACU, there was no difference in cumulative OME use in the first 24 hours (95% CI -9.745-24.10, p-value 0.4021).

Conclusion

In this study, we demonstrated that ESP blocks are an effective regional anesthesia technique to reduce postoperative pain and opioid consumption. The ESP block can serve as a useful and safe alternative to either thoracic epidural or paravertebral block techniques in thoracic and upper abdominal surgeries for perioperative pain management.

## Introduction

The treatment of pain is an important part of the perioperative period. Postoperative pain not only causes anxiety and subjective discomfort but can also contribute to poor postoperative outcomes. This includes poor rehabilitation, late mobilization, increased opioid consumption, and longer hospital length of stay [1]. Ultrasound-guided erector spine plane (ESP) block is a relatively novel and widely used regional anesthetic technique for the treatment of perioperative analgesia. It was first described for the treatment of thoracic neuropathic pain in 2016 [2] and has gained considerable interest from both clinical and academic circles due to its straightforward application, effectiveness, and safety profile across various clinical situations [3].

The ESP block is a paraspinal fascial plane block that can be implemented as either a single shot or a catheter for continuous infusions [4]. It is performed by using ultrasound to guide the injection of local anesthetic into the myofascial plane that is deep to the erector spinae muscle but superficial to the transverse process on which the muscle attaches [3,5]. The local anesthetic is thought to spread within this potential space and diffuse into abutting structures [3,6], such as the paravertebral and epidural spaces (containing spinal nerves, dorsal rami, and ventral rami) [7-9], lateral cutaneous nerves (contained by the serratus anterior and intercostal muscles) [10], and even the quadratus lumborum (at low thoracic and lumbar levels) [11].

Though it was first introduced for thoracic pain, the expansion of the ESP block for pain from the shoulder to the proximal lower extremity has become increasingly routine due to the extension of the erector spinae muscle and plane from the cervicothoracic to lumbar spine [3]. The level of the injection site should correspond to the spinal nerve territory that is desired for analgesia, but the fact that the local anesthetic from ESP blocks travels in both cephalad and caudad directions from the point of injection allows it to provide analgesic properties to multiple nerve territories [3,12]. This enables a considerable level of adaptability, particularly when the injection site needs to be far from the targeted area of pain relief [3,13,14].

One of the factors contributing to the widespread acceptance of the ESP block is its substantial safety margin. The needle is inserted superficially, allowing for the avoidance of the pleura, spinal cord, and major blood vessels. The transverse process is readily visible via ultrasound imaging and provides a distinct target, minimizing the risk of misplacement. The spread to the epidural space has been described but is a minimal amount of the total dose of the anesthetic, which results in a negligible to zero chance of sympathectomy or hypotension [3]. The biggest complication for clinicians to consider is the risk of local anesthetic systemic toxicity, a phenomenon that occurs when a large dose of local anesthetic is injected into an area of rich vascularization, such as the musculofascial plane [15]. The ESP block is a beneficial asset in regional anesthesia, especially when used for perioperative pain. The use of ESP blocks has been proven proficient in reducing postoperative opioid consumption and pain scores when compared with non-block care [16].

While the versatility, simplicity, and safety of the ESP block have been demonstrated [3], there is currently a gap in the anesthesia literature regarding its comparison between thoracic and abdominal surgeries. This retrospective study compared the efficacy of the ESP block in postoperative analgesia in two distinct groups: those who underwent gastric sleeve surgery and those who underwent bilateral breast surgery.

## Materials and methods

The patient population for this IRB-approved retrospective cohort study was obtained by searching the acute pain service records at a tertiary care academic center from July 2018 to December 2022. This retrospective study included 50 patients in the non-cardiac thoracic surgery group (bilateral breast mastectomy with reconstruction) and 50 patients in the abdominal surgery group (robotic or laparoscopic sleeve gastrectomy). The ESP block requires access to the patient’s back and was performed in the prone position. The ESP block was performed prior to surgery to take advantage of intraoperative opioid-sparing effects. We used a curved-array ultrasound transducer (2-5 MHz) and a 21G x 100 mm needle to perform an in-plane ESP block under ultrasound guidance. We performed single injection bilateral ESP blocks at T3-5 levels for the mastectomy group and T8-10 levels for sleeve gastrectomy patients. We used 25 ml of 0.25% ropivacaine for each side ESP block. Following intravenous access, application of appropriate monitors, and skin disinfection, the ultrasound transducer is placed in a parasagittal orientation over the tips of the transverse process at the desired target intervertebral level. All patients were given 1 mg of midazolam and 50 mcg of fentanyl before the block placement for comfort. The skin at the insertion point was injected with 2-3 ml of 1% lidocaine. The block needle was inserted in-plane to the ultrasound transducer in either a cranial-to-caudal or caudal-to-cranial direction; the choice should be dictated by ergonomics and the desired direction of spread. The transverse process is contacted gently with the needle tip and local anesthetic (0.25% ropivacaine) injected. This is signaled by a linear pattern of fluid spread in both cranial and caudal directions that separates and lifts the erector spinae muscle off the transverse process.

All patients received bilateral ESP blocks, performed under ultrasound guidance. Various parameters were evaluated including oral morphine equivalents (OMEs) and visual analog scale (VAS) scores during Post-anesthesia care unit (PACU), 6, 12, and 24-hours postop. The use of abortive antiemetic medications within 24 hours was also measured to evaluate the incidence of nausea and vomiting. The results were analyzed and compared. No control group is included, as all patients at our institution receive a peripheral nerve block as a part of the institution's enhanced recovery pathway (ERP).

Statistical analysis and data presentation 

Data are presented as the mean and standard error of the mean for continuous variables or the number and percentage of total for categorical variables. Group comparisons were conducted using T-tests (OME 24-hour cumulative, number of antiemetics used) or two-way ANOVA with Sidak’s multiple comparison test for time course data (OME, VAS). A p-value of less than 0.05 was considered statistically significant. All statistical analyses and graph generation were carried out using Graphpad Prism version 10 for Mac OS X (Graphpad, Boston, MA).

## Results

Our retrospective study compared two distinct patient groups. The first group underwent gastric sleeve surgery and received an abdominal ESP block and the second group undergoing breast surgery received a thoracic ESP block for postoperative analgesia. This data was extracted by chart review from the electronic medical record (EMR). The patient demographics are listed in Table 1.

**Table 1:** Demographic information by group. ASA: American Society of Anesthesiologists Physical Status Classification System; BMI: Body mass index; ESP: Erector spinae plane block; SE: Standard error of the mean; N=Number.

Characteristic	Abdominal ESP (n = 50)	Thoracic ESP (n = 50)
Age (years), mean (SE)	45.2 (1.4)	55.5 (1.9)
Race/ethnicity, N (%)		
-African American	34 (68%)	12 (24%)
-American Indian	0(%)	0 (0%)
-Asian	0 (%)	0 (0%)
-White	16 (32%)	38 (76%)
Sex, N (%)		
-Female	45 (90%)	50 (%)
-Male	5 (5%)	0 0(%)
BMI, mean (SE)	50.5 (1.3)	28.6 (1.2)
ASA, mean (SE)	3.1 (0.05)	2.7 (0.06)

Primary outcome measures for this study were the amount of oral morphine equivalents (OMEs) used throughout the first 24 hours and cumulatively in the first 24 hours. Secondary outcomes included visual analog pain scale (VAS) scores during admission and level of nausea/vomiting assessed by the number of times antiemetic medication had to be administered in the first 24 hours. Outcome measures for the two groups (abdominal ESP and thoracic ESP) are summarized in Table 2.

**Table 2:** Study outcomes by group. ESP: Erector spinae plane block; N: Number; OME: Oral morphine equivalent; PACU: Post-anesthesia care unit; SE: Standard error of the mean; VAS: Visual analog scale. P-value from Sidak multiple comparisons following two-way analysis of variance (OME, VAS) or T-test (OME 24-hour cumulative, number of antiemetics used).

Outcome	Abdominal ESP (n = 50)	Thoracic ESP (n = 50)	P-value
OME, mean (SE)			
-PACU	36.5 (28.5)	23.1 (3.5)	0.0003
-6 hours	6.5 (1.9)	11.5 (1.6)	0.4411
-12 hours	3.8 (1.0)	8.3 (1.4)	0.2546
-24 hours	6.7 (1.7)	18.1 (2.1)	0.0027
-24 hours (cumulative)	53.3 (5.9)	60.5 (6.2)	0.4021
Number of antiemetic administered, N (SE)	2.3 (0.25)	0.86 (0.17)	<0.0001
VAS pain scale, mean (SE), maximum			
-PACU	6.5 (0.5), 10	5.2 (0.4), 10	0.0443
-6 hours	3.2 (0.4), 8	3.1 (0.4), 8	0.8852
-12 hours	2.9 (0.4), 9	2.3 (0.4), 10	0.3568
-24 hours	2.9 (0.4), 9	2.7 (0.4), 7	0.6868

**Figure 1:**
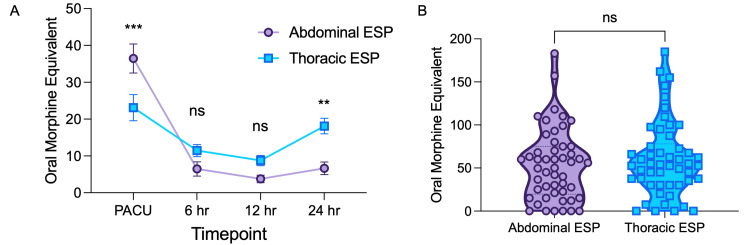
(A) Line diagram for OME use over time by group. (B) Violin plot for cumulative OME use for first 24 hours postop. ESP: Erector spinae plane block; OME: Oral morphine equivalents; PACU = Post-anesthesia care unit; ** denotes P ≤ 0.01; *** denotes P ≤ 0.001; ns= not significant; P>0.05.

To evaluate the analgesic efficacy of abdominal and thoracic ESP blocks, we performed an analysis of the electronic Medication Administration Record (MAR) to examine the distribution of pro re nata (PRN) opioid medication over time (Figure 1). OMEs were calculated for patients during their stay in the PACU and at 6, 12, and 24 hours postoperatively.

Two-way analysis of variance (ANOVA) was performed to estimate how the mean OME changed according to block type by timepoint postoperatively. Simple main effects analysis showed that postoperative timepoint had a statistically significant effect on OME (P<0.0001), accounting for 22.1% of the total variation. Additionally, simple main effect analysis showed that the individual patient significantly influenced OME (P<0.0001), accounting for 29.4% of the total variation. Though simple main effects analysis for ESP block type did not have a statistically significant effect on OME (P=0.3452), accounting for 0.27% of the total variation, there was a statistically significant interaction between the effects of ESP block type (abdominal vs thoracic) and postoperative timepoint (F(3,294) = 12.94, P<0.0001), accounting for 5.6% of total variation.

To compare OME usage at different timepoints for the abdominal and thoracic ESP groups, we conducted Sidak’s multiple comparison test post hoc. The mean difference (MD) in OME consumption for the abdominal ESP group compared to the thoracic ESP group was statistically significantly different in the PACU (mean difference 13.35 OME, 95% CI 4.97-21.73, P-0.0003). There was no difference in OME consumption at 6 hours (mean difference abdominal ESP- thoracic ESP -5.005, 95% CI -13.38-3.372, P=0.4420) or 12 hours (mean difference abdominal ESP- thoracic ESP 11.47, 95% CI -13.37- 3.387, P=0.4450). At 24 hours postoperatively, the mean difference in OME consumption for the abdominal ESP group compared to the thoracic ESP group was statistically significantly different (mean difference -11.47 OME, 95% CI -19.85- -3.093, P-0.0027) (Figure 1A).

Despite the abdominal ESP group having more OME utilization in the PACU and the thoracic ESP group having more OME utilization at 24 hours, there was no difference in cumulative OME use in the first 24 hours (mean difference abdominal ESP group compared to the thoracic ESP group 7.175 OME, 95% CI -9.745-24.10, P=0.4021) (Figure 1B).

**Figure 2:**
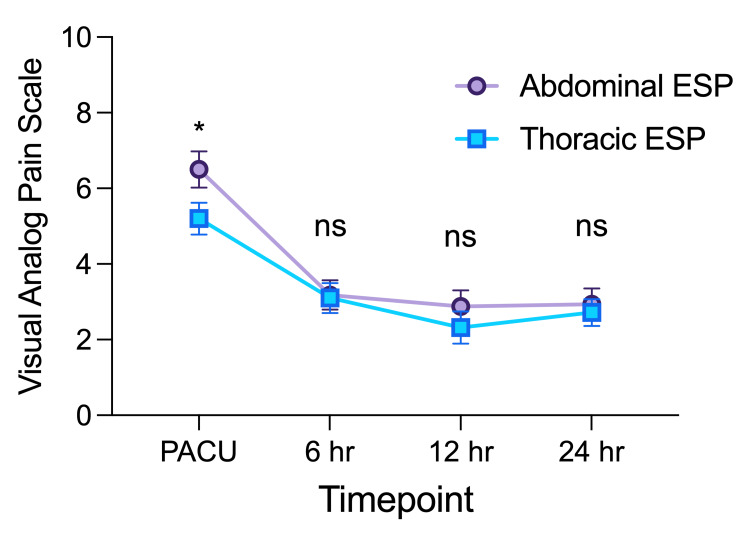
Pain scores over time by group. ESP: Erector spinae plane block; PACU: Post-anesthesia care unit; ns: not significant; P>0.05; * denotes P ≤ 0.05.

Visual analog scale (VAS) pain scores were tracked longitudinally up to 24 hours postoperatively following either sleeve gastrectomy in the abdominal ESP group or breast surgery in the thoracic ESP group (Figure 2).

Two-way analysis of variance (ANOVA) was performed to estimate how the mean VAS pain score changed according to block type by timepoint postoperatively. Simple main effects analysis showed that postoperative timepoint had a statistically significant effect on VAS (P<0.0001), accounting for 16.7% of total variation. Additionally, simple main effect analysis showed that the individual patient significantly influenced VAS (P<0.0001), accounting for 34.25% of the total variation. Simple main effects analysis for ESP block type did not have a statistically significant effect on VAS (P=0.1571), accounting for 0.71% of the total variation. There was not a statistically significant interaction between the effects of ESP block type (abdominal vs thoracic) and postoperative timepoint (F(3,294) = 1.1, P=0.3432), accounting for 0.54% of total variation.

To compare VAS usage at different timepoints for the abdominal and thoracic ESP groups, we conducted Sidak’s multiple comparison test post hoc. The mean difference in VAS pain score for the abdominal ESP group compared to the thoracic ESP group was statistically significantly different in the PACU (mean difference 1.3 VAS, 95% CI 0.03-2.56, P-0.0443). There was no difference in VAS consumption at 6 hours (mean difference abdominal ESP- thoracic ESP 0.08, 95% CI -1.02-1.12, P=0.8852), 12 hours (mean difference abdominal ESP- thoracic ESP 0.56, 95% CI -0.64-1.76, P=0.3568), or 24 hours (mean difference abdominal ESP: thoracic ESP 0.22, 95% CI -0.86-1.3, P=0.6868) (Figure 2).

**Figure 3:**
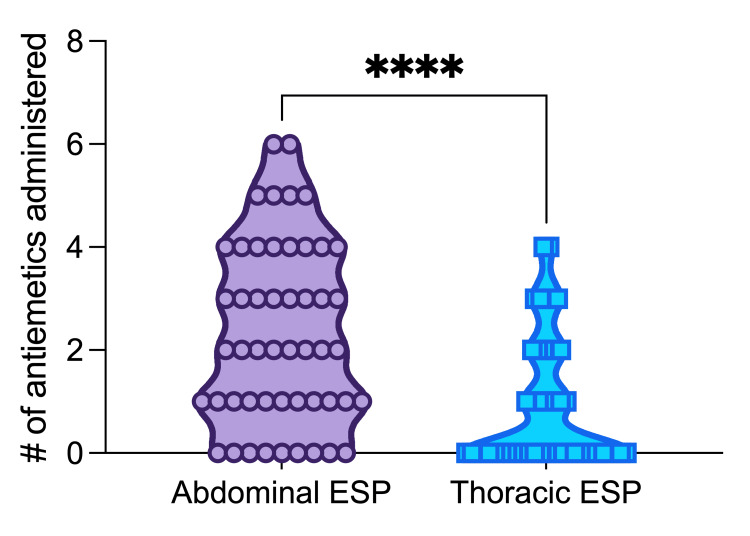
Antiemetic use by group. ESP: Erector spinae plane block; **** denotes P ≤ 0.0001.

We assessed whether there was a difference in nausea/vomiting profile by quantifying the number of times an antiemetic medication was administered in the first 24 hours postoperatively between the abdominal ESP group and thoracic ESP group. The abdominal ESP group required significantly more antiemetic pharmacotherapy compared to the thoracic ESP group (mean difference 1.4 antiemetics administered, 95% CI 0.84-2.04, P<0.0001) (Figure 3).

## Discussion

Postoperative pain has been associated with poor patient outcomes, such as decreased rehabilitation, respiratory complications, longer hospital admissions, and poor quality of life [17]. Because up to 10% of opioid-naïve patients were seen to have persistent opioid use after various surgeries, there has been an effort to optimize multi-modal agents and regional anesthesia practices as a measure against postoperative pain [18]. For the management of laparoscopic surgeries, local anesthetic infiltration at port sites was not shown to improve postoperative pain outcomes, but ESP blocks have been associated with lower pain scores and decreased opioid use [19,20]. 

Erector spinae plane block has been used as an intervention for providing postoperative analgesia in patients undergoing bariatric and metabolic surgeries. In a recent systematic review, the ESP block was compared to the control (no block) group for the primary outcome was 24-hour opioid consumption and the secondary outcomes were intraoperative opioid use, pain scores, time to rescue analgesia, and complications [21]. The 24-hour opioid consumption was significantly lesser in the ESP group when compared to the control (mean difference {MD}: −10.67; 95% confidence interval {CI}: −21.03, −0.31). The time to rescue analgesia was significantly more in the ESP group (MD: 114.36; CI: 90.42, 138.30). The review concluded that bilateral ESP blocks provide opioid-sparing analgesia and better pain scores when compared to control. These results should be interpreted with caution due to high heterogeneity and demographic variability among the included studies.

Our retrospective study found that the use of ESPs in thoracic and abdominal surgeries for the treatment of postoperative pain was equivalent. There is no control group since all patients receiving gastric sleeve and breast surgery receive a nerve block as a part of our institution’s ERP. Because of the types of surgeries compared, the patient population between groups is statistically different for age, BMI, and ASA. Because the typical demographic that undergoes gastric sleeve surgery includes obese patients, one would expect an increased BMI and ASA in this group. Similarly, the mastectomy group in this study only includes the female gender which may influence study outcomes.

Although the ESP block is described as a fascial plane block, its exact mechanism is widely debated with theories including local spread of local anesthetic, diffusion to neural structures, and epidural spread [3,6]. Also, the anatomical coverage appears to vary by study. While some have shown the ability of local anesthetic to diffuse to both thoracic dorsal and ventral rami via dye on cadaver studies and clinically provide visceral coverage in abdominal surgeries [2,22], others suggest the ESP only provides adequate coverage for pain control to the posterior chest wall but spares the anterior-lateral chest walls and abdomen [23]. Biovicini et al reported the effective use of bilateral ESP blocks for breast reconstructive surgery and suggested it to be a comparable alternative to paravertebral block (PVB) and thoracic epidural (TEA) techniques [24].

Gürkan et al. reported the efficacy of ESP block as compared to PVB for postoperative analgesia in breast surgery [25]. In this study, opioid consumption was 5.6 ± 3.43 mg in the ESP group, 5.64 ± 4.15 mg in the PVB group, and 14.92 ± 7.44 mg in the control group. Our study found that although patients in the thoracic group had significantly lower OME consumption and higher pain scores in PACU, there was not a statistically significant difference in cumulative OMEs in 24 hours (Figure 1). Because patients in the thoracic arm of this study underwent bilateral mastectomies with reconstruction, coverage of the anterolateral and anteromedial branches of thoracic intercostal nerves T3-T5 would be needed to effectively block sensory innervation of the breast and provide appropriate pain control [26]. The clinical and demographic variability in ESPs to adequately cover these anatomical regions, as described in previous literature, likely contributed to the results in OME consumption.

Patients in the abdominal surgery group required significantly more anti-emetic medications within the first 24 hours postop. However, the elevated use of these medications does not correspond to OME consumption. Although this group had a significantly higher OME consumption in PACU, the OME utilization decreased and was lower than the thoracic group at 6, 12, and 24 hours. The increased incidence of nausea and vomiting in the abdominal group is likely a result of the type of surgery and patient demographic, as obese patients undergoing laparoscopic bariatric surgery have been associated with increased risk for postoperative nausea and vomiting (PONV) [27].

The clear limitation of this study is variability in the study population and extent of surgery type. The demographic variability and duration of surgery may influence pain-related study outcomes. The nature of this study as a retrospective analysis limits the ability to control for other variables that could contribute to postoperative pain scores. The blocks were not immediately assessed before surgery to identify failed blocks early on. Sensory testing could be done before surgery to find out dermatomal distribution and adequacy of block analgesia. Future studies need to be done to take into account the ERP protocol of the institution, preoperative sedation, intraoperative pain control, and the use of multimodal agents in the perioperative setting.

## Conclusions

The erector spinae plane (ESP) block provides a safe, effective, and relatively easy-to-perform regional technique that can be utilized for an enhanced recovery pathway. In this study, we demonstrated that ESP blocks are an effective regional anesthesia technique to reduce postoperative pain and opioid consumption. The ESP block can serve as a useful and safe alternative to either thoracic epidural or paravertebral block techniques in thoracic surgeries for perioperative pain management. Future studies are warranted to examine the effect of additives or liposomal bupivacaine on prolonging single ESP blocks.
